# Supplementary material for: Zbtb11 interacts with Otx2 and patterns the anterior neuroectoderm in Xenopus
Source: PLoS One. 2024 Jul 31;19(7):e0293852. doi: 10.1371/journal.pone.0293852 (PMC11290676; doi:10.1371/journal.pone.0293852)
Supplement: S1 Fig — (A) Temporal expression of zbtb11.L and zbtb11.S in X. laevis embryos. Expression levels (transcripts per million: TPM) are calculated from RNA-sequencing (RNA-seq) datasets of X. laevis developing embryos (Session et al., 2016). (B) Temporal expression of zbtb11 in X. tropicalis embryos. Expression levels (transcripts ×1000) are calculated from RNA-seq datasets of X. tropicalis developing embryos (Owens et al., 2016). Images are generated using Xenbase (http://www.xenbase.org/) and developmental stages (oocyte and Nieuwkoop-Faber [NF] stages) are as indicated (A,B). (PDF) [file pone.0293852.s001.pdf]

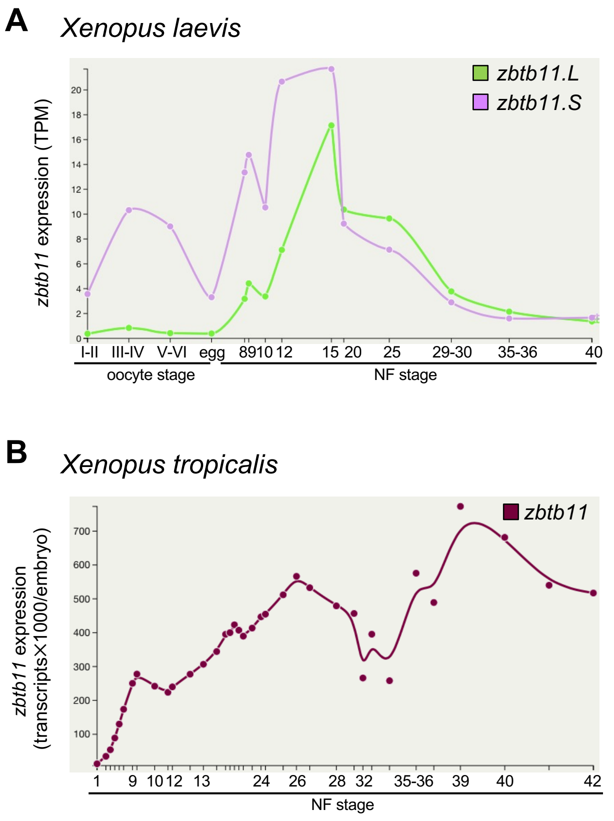

S1 Fig. Developmental expression of *zbtb11* in *Xenopus laevis* and *Xenopus tropicalis*.

(A) Temporal expression of *zbtb11.L* and *zbtb11.S* in *X. laevis* embryos. Expression levels (transcripts per million: TPM) are calculated from RNA-sequencing (RNA-seq) datasets of *X. laevis* developing embryos (Session et al., 2016). (B) Temporal expression of *zbtb11* in *X. tropicalis* embryos. Expression levels (transcripts ×1000) are calculated from RNA-seq datasets of *X. tropicalis* developing embryos (Owens et al., 2016). Images are generated using Xenbase (http://www.xenbase.org/) and developmental stages (oocyte and Nieuwkoop-Faber [NF] stages) are as indicated (A,B).
