# Supplementary material for: Zbtb11 interacts with Otx2 and patterns the anterior neuroectoderm in Xenopus
Source: PLoS One. 2024 Jul 31;19(7):e0293852. doi: 10.1371/journal.pone.0293852 (PMC11290676; doi:10.1371/journal.pone.0293852)
Supplement: S2 Fig — Alignment of Zbtb11 amino acid sequences between Xenopus laevis (Xl), Xenopus tropicalis (Xt), Homo sapiens (Hs), Mus musculus (Mm), and Danio rerio (Dr). Xenopus laevis has two homeologs: L and S genes. Light blue boxes, the conserved regions CR1, CR2 and CR3; purple box, the integrase-like histidine-histidine-cysteine-cysteine (HHCC) motif; brown box, the BTB domain; magenta boxes, C2H2 type zinc fingers. The blue-coloured cysteine residue (C116) indicates a mutation site in the neutrophil-deficient zebrafish mutant (marsanne, mne). Red histidine residues (H729 and H880) indicate ZBTB11 mutation sites that are associated with intellectual disability. Arrows indicate the end of the Venus-BTB construct, and the beginning of the Venus-Znf construct. (PDF) [file pone.0293852.s002.pdf]

S2 Fig. (Satou-Kobayashi et al.)

```
Xl_Zbtb11.S    MSSEESYLAILRYLTNEREPYAPGTEGNAKRKIRKAAACYVVRAGTLYYQRKLRDKEGFT 60
Xl_Zbtb11.L    MSSEESYLAILRYLTNEREPYAPGTEGNAKRKIRKAAACYVVREGTLYYQRKLRDKEGFT 60
Xt_Zbtb11      MSNEESYLAILRYLTNEREPYAPGTEGNAKRKIRKAAACYVVRAGTLYYQRKLRDKEGFT 60
Hs_Zbtb11      MSSEESYRAILRYLTNEREPYAPGTEGNVKRKIRKAAACYVVRGGTLYYQRRQRHRKTFA 60
Mm_Zbtb11      MSSEESYRAILRYLTNEREPYAPGTEGNVKRKIRKAAACYVVRGGTLYYQRRQRHRKTFA 60
Dr_Zbtb11      MSSEESYLAIQRYLTDEREPYAPGTHGNTKRKIRKAAACYIVRNGILYYQRRQKGLDEFT 60
               **.**** ** ****:*********.**.***********:** * *****: :  . *:

Xl_Zbtb11.S    ELEVVLQLERRKRLIEAAHVGTGGEHLTRHQTWHVLSQSYWWRGILKHMKDYIKDCSKCQ 120
Xl_Zbtb11.L    ELEVVLQPERRKGLIEAAHINTGGEHLTRHQTWHVLSQSYWWRGILKHMKDYIKDCSKCQ 120
Xt_Zbtb11      ELEVVLQPERRKGLIEAAHIGTGGEHLTRHQTWHVLSQTYWWRGILKHMKDYIKDCSKCQ 120
Hs_Zbtb11      ELEVVLQPERRRDLIEAAHLGPGGTHHTRHQTWHYLSKTYWWRGILKQVKDYIKQCSKCQ 120
Mm_Zbtb11      ELEVVLQPERRQGLIEAAHLGPGGTHHTRHQTWHDLSKTYWWRGILKQVKDYIKQCSKCQ 120
Dr_Zbtb11      ELEVVLTADRRKELITEAHITSGGEHLNQQQTWEIISQKYWWRGVLKQVKDCIKECIHCQ 120
               ******  :**: **  **: .** * .::***. :*:.*****:**::** **:* :**

Xl_Zbtb11.S    ERTDRSRPSNDASEMLEEPGLDVRS--DTNETDDESSNPASNTSHTPKPSRKKPTTKHEL 178
Xl_Zbtb11.L    ERTDRSRSTNDASEMLEELGLDARS--DTNETDDESSNPASNTSHTPKTSRKKPTAKHEL 178
Xt_Zbtb11      ERTDRSRSTNDASEMLEELGLDVRS--DTNETDDESSNPASNTYQTPKPARKKPTAKHEL 178
Hs_Zbtb11      EKLDRSRPISDVSEMLEELGLDLESGEESNESEDDLSNFTSSPTTASKPAKKKPVSKHEL 180
Mm_Zbtb11      EKLDRSRPISDASEMLEELGLDLDSGEESNESEDDLSNFTSPPSTASKSSKKKPVSKHEL 180
Dr_Zbtb11      TKQEKGR--NNTEDAPKQTARQVRQKISSACASEEEDD---EIHEDLEEEQSTTVDKHEL 175
                : ::.*  .:..: :: . :  .  .:  :.:: .:            :  :.... ****

Xl_Zbtb11.S    VFVDSKGIVKQASSKHGQTILDQLNQQRLNNQFCDVTLLIEGQEFKAHKAVLAASSEYFR 238
Xl_Zbtb11.L    VFVDSKGIVKQTSSKHGQTILNQLNQQRLNNQFCDVTLLIEGQEFKAHKAVLAASSEYFT 238
Xt_Zbtb11      VFVDSKGIVKQSSSKHGQTILDQLNQQRLNNQFCDVTLLIEGQEFKAHKAVLAASSEYFR 238
Hs_Zbtb11      VFVDTKGVVKRSSPKHCQAVLKQLNEQRLSNQFCDVTLLIEGEEYKAHKSVLSANSEYFR 240
Mm_Zbtb11      VFVDTKGVVKRSSPKHCQAVLKQLNEQRLSNQFCDVTLLIEGEEYKAHKSVLSANSEYFR 240
Dr_Zbtb11      VFVDSKGIVKQFLQKHGQTMLDKLNLQRMNNEFCDITLIIEGEEYRAHKAVLASCSDYFY 235
               ****:**:**:   ** *::*.:** **:.*:***:**:***:*::***:**:: *:**

Xl_Zbtb11.S    ELFIEKGAVSSHEAFVDLSGFCKSSFLPLLEFAYTSELIFDFCSMAEISMLARHLFMNEV 298
Xl_Zbtb11.L    ELFIEKGAVTSHEAVVDLSGFCKSSFLPLLEFAYTSELVFDFCSMAEISMLARHLFMNEV 298
Xt_Zbtb11      ELFIEKGAVSSHEAFVDLSGFCKSSFLPLLEFAYTSELVFDFCSMAEISMLARHLFMNEV 298
Hs_Zbtb11      DLFIEKGAVSSHEAVVDLSGFCKASFLPLLEFAYTSVLSFDFCSMADVAILARHLFMSEV 300
Mm_Zbtb11      DLFIEKGAVSSHEAVVDLSGFCKASFLPLLEFAYTSVLSFDFCSMADVAVLARHLFMSEV 300
Dr_Zbtb11      ELFVEKGAVSSHEAVVDLSGFSKASFLPLLDFAYTSNLTFNFCVMAEVATLARHLLMAEV 295
               :**:*****:****.******.*:******:***** * *:** **::: *****:* **

Xl_Zbtb11.S    LEICESVHRQMETNQINLCQKVDVVSENSLLDASVVKLQAVGNNAPPLLAEVINSQKTSL 358
Xl_Zbtb11.L    LEICESVHRQMETNQINLCQKVDVVSESSCLDGRVVELQTVGINAPPLLAEVITSQKTSL 358
Xt_Zbtb11      LEICESVHKQMETNQINLCQKVDVVSENGLLDGRVVKLQAVGNNAPQLLAEVINSQKTSL 358
Hs_Zbtb11      LEICESVHKLMEEKQLTVYKKGEVQT--------------------------VASTQDLR 334
Mm_Zbtb11      LEICESVHKLMEEKQLTVYKKGEVQT--------------------------VASTQDLA 334
Dr_Zbtb11      LQICESVHKKVEEQKLMVYQRGDIHT------------------VLPNQPASTQPITPTA 337
               *:******: :* ::: : :: :: :                          .

Xl_Zbtb11.S    SNNENLECETVATETVTNSTDVAVSQLSEVPTVQLVTGPEVHISSLTGTAAIYLSDVSSE 418
Xl_Zbtb11.L    VNNKNVACEPVATETVTNSTDGVLSQLSEEPTVHLVTSPEVPISSLTGMTAIYLSDMSSE 418
Xt_Zbtb11      VNNENVECEAAATETVTNSTDGAISQSSEESTVQLVTSPEIPISSLTGTTAIYLSDVSSE 418
Hs_Zbtb11      VQNGGTAPPVASSEGTTTS---LPTELGDCEIVLLVNG-ELPEAEQNGEV---------- 380
Mm_Zbtb11      AHNGTTTPPGTRNEATTT----LSGELGHCEIVLLVNG-ELPEAEQNGEP---------- 379
Dr_Zbtb11      AETYVVTMQSDGTAEAGQSLAVITSEIGTAESLALLAGATVDGETMTVVTHSGQAGSAES 397
                ..       .  .        : .    : *: .  :     .

Xl_Zbtb11.S    LQPAVIIPAVIDASNVQ-HQQILGDAPEDSTPADHMESQQPTGDNNIDASDKVQSVMFTT 477
Xl_Zbtb11.L    LQTAVIIPAVKDISNVHEHQQILSDTQEDSTPAEHSESPQSTGNGNMDASDKVQPVTLAT 478
Xt_Zbtb11      LQPAVIIPAVIDVSNVQEHQQILGDTPEDSTTAEHPESQQSTAGSNMDVPDEVLSVTCTT 478
Hs_Zbtb11      ------------------------GRQPEPQVSSEAESALSSVGCIADSHPEMESVDLIT 416
Mm_Zbtb11      ------------------------EQQPAPQASPEAEASVSPVEGIPEPHPEMGTASLAK 415
Dr_Zbtb11      LAMVAHS-----------------GQAEEGETMTLVTHSGQAGSGESLAVVQACWSVEEPQ 441
                                          :   .:  .          ..

Xl_Zbtb11.S    SEKKLCAEEPQPKDVSLKEITNSNTNGDPQISVAVAEESSASVIDLETTCAEDVVIGNTD 537
Xl_Zbtb11.L    SENKLPADDPQPKDVCLKEFAYSSTNGDPQISVSVAEEPSISVIDLETTCAEDVVIGNTD 538
Xt_Zbtb11      TEEKLPADDPQPKDVSMKELINSSTNGDPQVSVPVAEESSISVIDLETTCAEDVVIGNTD 538
Hs_Zbtb11      KNN-------QTE---------LETSNNRENNTVSNIHPKLSKENVISSSPEDSGMGNDI 460
Mm_Zbtb11      ESN-------QPE---------SAVT--REDGIVASVHPKISKENVTNASQEDSDTGNDT 457
Dr_Zbtb11      VGDTPVVQSMQTG-AFLISVDPGKTGASEIVHLAAAAPPAVQDEPQASKPIHQTEPPAAA 500
                .       *.          .         .  .     .   .:

                                   Venus-BTB construct --->|
Xl_Zbtb11.S    PEP-----KVKIGEDPKRKRGRPHKSP--EDIENSSKKTRQILQSGEKCTYKGK--LRDC 588
Xl_Zbtb11.L    PEQ-----EFKS-EVPKRKRGRPRKSP--EDVEKSSKETKETLPSDDKCKYKGK--LRDC 588
Xt_Zbtb11      PDP-----EAKIGEVPKRKRGRPRKSP--EDVEKSCKETRETLPSGGKCTYKGK--LRDC 589
Hs_Zbtb11      SAE-----DICAEDIPKHRQ-KVDQPL--KDQENLVASTAKTDFGPDDDTYRSR--LRQR 510
Mm_Zbtb11      SPE-----DIGAKDCPDHSQ-SPGQPS--KDEDTLTEATEKTDSGPDDDTYRSR--LRQR 507
Dr_Zbtb11      PQPSPAPLKRRPGRPPKVKQQEPPPPLPSEDEPMEMEGVEDDEKQEDECIDPNKRYLRKR 560
               .       .      *.  :      .   :*        . .       .    .:  **.
```

S2 Fig. (Satou-Kobayashi et al.)

```
Xl_Zbtb11.S      LVGKGGYIRLHQGQG----------------KTLKKR-------KTNP-----KSAVQQA 620
Xl_Zbtb11.L      LVGTGGYIKLHKGQG----------------KPLKKR-------KTNP-----KSAVQQA 620
Xt_Zbtb11        LVGKGGYIMLHRGQE----------------KALKKR-------KANP-----KSAVQQA 621
Hs_Zbtb11        SVNEGAYIRLHKGME----------------KKLQKR-------KAVP-----KSAVQQV 542
Mm_Zbtb11        SVNEGGYIRLHKGME----------------KKLQKR-------KAIS-----KSAVQQV 539
Dr_Zbtb11        SVREGGYVRLHMGLETEEEEKITSPPAKIPQKGLAKRGRPVQSAKKNPETEFSETNLEAA 620
                  *  *.*: ** *                  * * **       *  .     :: :: .
                   |---> Venus-Znf construct

Xl_Zbtb11.S      AMKLLQRGKKKR--LVAPKKQIAE------------AQFKCDDCGLMFERRYALIMHSVK 666
Xl_Zbtb11.L      AMKLLQRGKKKR--LVAPKKQTAE------------AQFKCNDCGMMFQRRYALLMHSVK 666
Xt_Zbtb11        AMKLLQRGKKKR--LMVPKKQIAE------------AQFKCNDCGMMFQRRYALIMHSVK 667
Hs_Zbtb11        AQKLVQRGKKMKQPKRDAKENTEE------------ASHKCGECGMVFQRRYALIMHKLK 590
Mm_Zbtb11        AQKLVQRGKKMKQPKRDAKESTEE------------TAHKCGECGMVFPRRYAFIMHTLK 587
Dr_Zbtb11        PNAIVTSAKEPEPVIVEQPETVEEGALPGDPEGAVEGEHACNECGMVFQRRYTLIMHALK 680
                 .   :: .*: .       :   *              . *.:**::* ***:::** :*

Xl_Zbtb11.S      HEKSKEFKCPLCTKEFQYRASLRAHLIRHSKTGK---SEVNVVETEG-----DTVKGGTK 718
Xl_Zbtb11.L      HEKSKEFKCPLCTKEFQYSASLRAHLIRHSKTGK---AEVNVVETET-----DTVKGGTK 718
Xt_Zbtb11        HEKSKEFKCPLCTKEFQYRASLRAHLIRHSKTGK---AEVNVVETEA-----DTAKGGTK 719
Hs_Zbtb11        HERARDYKCPLCKKQFQYSASLRAHLIRHTRKDAPSSSSSNSTSNEASGT--SSEKGRTK 648
Mm_Zbtb11        HERARDYKCPLCKKQFQYSASLRAHLIRHTRKEAPTSSSSNSTSTEASGG--SSEKGRTK 645
Dr_Zbtb11        HEKARIFKCSICNKEFQYAASLRAHLARHKHQKSQRASLTRAMATEDSQGSDDQARFRTR 740
                 **::: :**.:*.*:*** ******* **.:      :  .   .*      .  :  *:

Xl_Zbtb11.S      RQFICGICGRTLPKLYSLRIHMLKHTGVKPHACEICGKAFTHKHGLKMHQALHESHKQFK 778
Xl_Zbtb11.L      RQFICGICGRTLPKLYSLRIHMLKHTGVKPHACEICGKAFTHRHGLKMHQALHEAHKQFK 778
Xt_Zbtb11        RQFICGICGRTLPKLYSLRIHMLKHTGVKPHACEVCGKTFTHKHGLKMHQALHESHKQFK 779
Hs_Zbtb11        REFICSICGRTLPKLYSLRIHMLKHTGVKPHACQVCGKTFIYKHGLKLHQSLHQSQKQFQ 708
Mm_Zbtb11        REFICSICGRTLPKLYSLRIHMLKHTGVKPHACQVCGKTFIYKHGLKLHQSLHQSQKQFQ 705
Dr_Zbtb11        REFVCDICGKTMPKLYSLRIHMLNHTGVRPHSCKVCGKSFATKHSLKMHRALHDSLKRFH 800
                 *:*:*.***:*:***********:****:**:*::***:*  :*.**:*::**:: *:*:

Xl_Zbtb11.S      CDMCDKSFVTNRSLQEHVSTHTGESKYLCSVCGKPFHRASGLSKHLKKHKPKVETRGYHC 838
Xl_Zbtb11.L      CHMCDKSFVTNRSLQEHVSTHTGESKYLCSVCGKAFHRASGLSKHLKKHKPKVETRGYHC 838
Xt_Zbtb11        CDMCEKSFVTNRSLQEHVSTHTGESKYLCSVCGKAFHRASGLSKHLKKHKPKVETRGYHC 839
Hs_Zbtb11        CELCVKSFVTKRSLQEHMSIHTGESKYLCSVCGKSFHRGSGLSKHFKKHQPKPEVRGYHC 768
Mm_Zbtb11        CELCVKSFVTKRSLQEHMSIHTGESKYFCSICGKSFHRGSGLSKHLKKHQPKPEVRGYHC 765
Dr_Zbtb11        CTVCEKSFVTKRSLEEHTSIHTGESKYLCTTCGASFHRASGLSKHLKKHQPKPEVRSFHC 860
                 * :* *****:***:** * *******:*: ** .***.******:***:** *.*.:**

Xl_Zbtb11.S      TQCEKSFFELRDLQQHMNKHLGVKPFECEYCGKSYSWKKDWYSHVKSHSVKEPYRCSVCG 898
Xl_Zbtb11.L      TQCEKSFFELRDLQQHMNKHLGVKPFECEFCGKSYSWKKDWYSHVKSHSVTEPYRCSVCG 898
Xt_Zbtb11        TQCEKSFFELRDLQQHMNKHLGVKPFECEFCGKCYSWKKDWYSHVKSHSVTEPYRCSVCG 899
Hs_Zbtb11        TQCEKSFFEARDLRQHMNKHLGVKPFQCQFCDKCYSWKKDWYSHVKSHSVTEPYRCNICG 828
Mm_Zbtb11        TQCEKSFFEARDLRQHMNKHLGVKPFQCQFCDKCYSWKKDWYSHVKSHSVTEPYRCNICG 825
Dr_Zbtb11        SHCDKSFFEAKDLQQHMNKHLGLKPFQCQVCGKCYSWKKDWYSHVKSHSVAEPFRCNVCG 920
                 ::*:***** :**:********:***:*: *.*.**************** **:**.:**

Xl_Zbtb11.S      KEFYEKALYRRHVNKATHGKKGRTKLNFEKTCEHCGKVFTQFREYRRHVNNHEGVKPFEC 958
Xl_Zbtb11.L      KEFYEKALYRRHVNKATHGKKGRTKLNFEKACEHCGKIFTQFREYRRHVNNHEGVKPFEC 958
Xt_Zbtb11        KEFYEKALYRRHVNKATHGKKGRTKLNFEKACEHCGKIFTQFREYRRHVNNHEGVKPFEC 959
Hs_Zbtb11        KEFYEKALFRRHVKKATHGKKGRAKQNLERVCEKCGRKFTQLREYRRHMNNHEGVKPFEC 888
Mm_Zbtb11        KEFYEKALFRRHVKKATHGKKGRAKQNLERVCDQCGRKFTQLREYRRHMNNHEGVKPFEC 885
Dr_Zbtb11        KEFFEKALFRRHVKKATHGKKGRVKQNLERECEHCGRKFTQLREYRRHMNNHQGVKPFEC 980
                 ***:****:****:*********.* *:*: *::**: ***:******:***:*******

Xl_Zbtb11.S      LTCGVAWADARSLKRHVRTHTGERPYVCPVCNDGYIDARTLRKHMTKTHKDYVPGKIMLE 1018
Xl_Zbtb11.L      ITCGVAWADARSLKRHVRTHTGERPYVCPVCNEGYIDARTLRKHMTKTHKDYVPGKIMLE 1018
Xt_Zbtb11        LTCGVAWADARSLKRHVRTHTGERPYVCPVCNEGYIDARTLRKHMTKTHKDYVPGKIMLE 1019
Hs_Zbtb11        LTCGVAWADARSLKRHVRTHTGERPYVCPVCSEAYIDARTLRKHMTKFHRDYVPCKIMLE 948
Mm_Zbtb11        LTCGVAWADARSLKRHVRTHTGERPYVCPVCSEAYIDARTLRKHMTKFHRDYVPCKIMLE 945
Dr_Zbtb11        LTCGVAWADARSLKRHVRTHTGERPYVCPLCQEAHIDARTLRKHITKFHGDQLPGKIMLE 1040
                 :****************************:*.:.:*********:** * * :* *****

Xl_Zbtb11.S      KDSLQFHNQGTQVEHAVSILSAELDEPQAEIVT---EEIETVMVA------EEAMAQPPI 1069
Xl_Zbtb11.L      KDSLQFHNQGTQVEHAVSILSTELDEPQAEIVT---EEIETAVVA------EEAMVQALN 1069
Xt_Zbtb11        KDSLQFHNQGTQVEHAVSILSTDLDEPQAEIVT---EEIETVVVA------EEAMAQTPN 1070
Hs_Zbtb11        KDTLQFHNQGTQVAHAVSILTAGMQE-QESSGP---QELETVVVTGETMEALEAVAATEE 1004
Mm_Zbtb11        KDTLQFHNQGTQVEHAVSILTADMQE-QESSGP---QELETVVVTGETMEVLEAVAATEE 1001
Dr_Zbtb11        KDTLQFHNQGTQVEHAVSILSSELPPELQPPQPPATEEIETVLITEET---VEAVQAMND 1097
                 **:********** ******:: :        .   :*:**.:::       **:

Xl_Zbtb11.S      CTAVSSLSDQSIMQVVNYVLSQQQGQPITDVAEELETVEVEVGQISEVV 1118
Xl_Zbtb11.L      DTAVSSLSDQSIMQVVNYVLAQQQGQKMTDVSEELETVEVEVGQISEVV 1118
Xt_Zbtb11        STAVSSLSDQSIMQVVNYVLSQQQGQKIS--EEEIETVEVEVGQISEVV 1117
Hs_Zbtb11        YPSVSTLSDQSIMQVVNYVLAQQQGQKLSEVAEAIQTVKVEVAHISGGE 1053
Mm_Zbtb11        CPSVSTLSDQSIMQVVNYVLAQQQGQKLSEVAEAIQTVEVEVAHMPEAE 1050
Dr_Zbtb11        GAAVTTLSDQSIMQVVNYVLAQQAAVKVEEAPEIIQTMEVEVAHVAEVE 1146
                  .:*::**************:** .  :    * ::*::***.::.
```

S2 Fig. Alignment of Zbtb11 amino acid sequences between different species. Alignment of Zbtb11 amino acid sequences between *Xenopus laevis* (Xl), *Xenopus tropicalis* (Xt), *Homo sapiens* (Hs), *Mus musculus* (Mm), and *Danio rerio* (Dr). *Xenopus laevis* has two homeologs: L and S genes. Light blue boxes, the conserved regions CR1, CR2 and CR3; purple box, the integrase-like histidine-histidine-cysteine-cysteine (HHCC) motif; brown box, the BTB domain; magenta boxes, C2H2 type zinc fingers. The blue-coloured cysteine residue (C116) indicates a mutation site in the neutrophil-deficient zebrafish mutant (marsanne, mne). Red histidine residues (H729 and H880) indicate ZBTB11 mutation sites that are associated with intellectual disability. Arrows indicate the end of the Venus-BTB construct, and the beginning of the Venus-Znf construct.
