# Supplementary material for: Zbtb11 interacts with Otx2 and patterns the anterior neuroectoderm in Xenopus
Source: PLoS One. 2024 Jul 31;19(7):e0293852. doi: 10.1371/journal.pone.0293852 (PMC11290676; doi:10.1371/journal.pone.0293852)
Supplement: S3 Fig — mRNA for Myc-Zbtb11, Venus-Zbtb11, or its deletion constructs was injected into two blastomeres at the 2-cell stage. Lysates were prepared at stages 10–10.5, and western blotting was performed with antibodies as indicated. (A) Western blotting of Myc-Zbtb11. SDS-polyacrylamide gel electrophoresis (PAGE) was carried out with a 7.5% polyacrylamide gel. (B) Western blotting of Venus, Venus-BTB, Venus-Znf, and Venus-Zbtb11. SDS-PAGE was carried out with a 10% polyacrylamide gel. Black arrowheads, nascent proteins (undegraded products). Calculated molecular weights (MW) of Zbtb11 constructs are as indicated besides dotted lines: Myc-Zbtb11, 140; Venus-Zbtb11, 160; Venus-BTB, 91; Venus-Znf, 87; Venus, 30. Brackets indicate degradation products, and the corresponding degradation products in the different constructs are indicated by the same colour (orange, magenta, or blue) and number of dots. White dots indicate non-specific bands. Molecular masses of protein size marker (kDa) are indicated on the right side. Uninj., uninjected sample. (PDF) [file pone.0293852.s003.pdf]

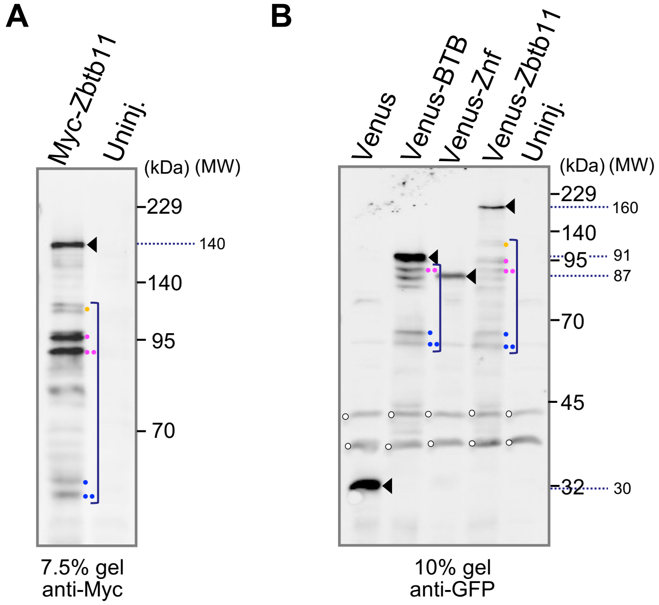

S3 Fig. Stability of Myc-Zbtb11, Venus-Zbtb11, and its deletion constructs in the *Xenopus laevis* embryo.

mRNA for Myc-Zbtb11, Venus-Zbtb11, or its deletion constructs was injected into two blastomeres at the 2-cell stage. Lysates were prepared at stages 10–10.5, and western blotting was performed with antibodies as indicated. (A) Western blotting of Myc-Zbtb11. SDS-polyacrylamide gel electrophoresis (PAGE) was carried out with a 7.5% polyacrylamide gel. (B) Western blotting of Venus, Venus-BTB, Venus-Znf, and Venus-Zbtb11. SDS-PAGE was carried out with a 10% polyacrylamide gel. Black arrowheads, nascent proteins (undegraded products). Calculated molecular weights (MW) of Zbtb11 constructs are as indicated besides dotted lines: Myc-Zbtb11, 140; Venus-Zbtb11, 160; Venus-BTB, 91; Venus-Znf, 87; Venus, 30. Brackets indicate degradation products, and the corresponding degradation products in the different constructs are indicated by the same colour (orange, magenta, or blue) and number of dots. White dots indicate non-specific bands. Molecular masses of protein size marker (kDa) are indicated on the right side. Uninj., uninjected sample.
