# Supplementary material for: Zbtb11 interacts with Otx2 and patterns the anterior neuroectoderm in Xenopus
Source: PLoS One. 2024 Jul 31;19(7):e0293852. doi: 10.1371/journal.pone.0293852 (PMC11290676; doi:10.1371/journal.pone.0293852)
Supplement: S4 Fig — (A) Fluorescence observation of Venus-NLS and Venus-Zbtb11. mRNA for Venus-NLS or Venus-Zbtb11 was injected into any one of the blastomeres at the 4-cell stage and fluorescence was observed at stages 14, 19–20, and 28–30. There are no differences in the fluorescence intensity between injected regions, indicating that the protein stability of Venus-Zbtb11 does not depend on tissue type. Merged, fluorescence images merged with bright-field images; Venus, fluorescence images. (B,C) Comparison of protein expression levels between Venus-NLS and Venus-Zbtb11. mRNA for Venus-NLS or Venus-Zbtb11 was injected into both blastomeres at the 2-cell stage. Lysates were prepared at stages 10, 16, and 19 and subjected to western blotting using anti-GFP and anti-β-tubulin antibodies. (B) Western blotting of Venus-NLS and Venus-Zbtb11. Upper panels, bands of Venus-NLS and Venus-Zbtb11; lower panels, bands of β-tubulin as a loading control. (C) Expression levels of Venus-NLS and Venus-Zbtb11. The relative expression level was obtained by dividing the band intensity of Venus-NLS or Venus-Zbtb11 by that of β-tubulin. Stages (st.) are indicated, respectively. (PDF) [file pone.0293852.s004.pdf]

S4 Fig. (Satou-Kobayashi et al.)

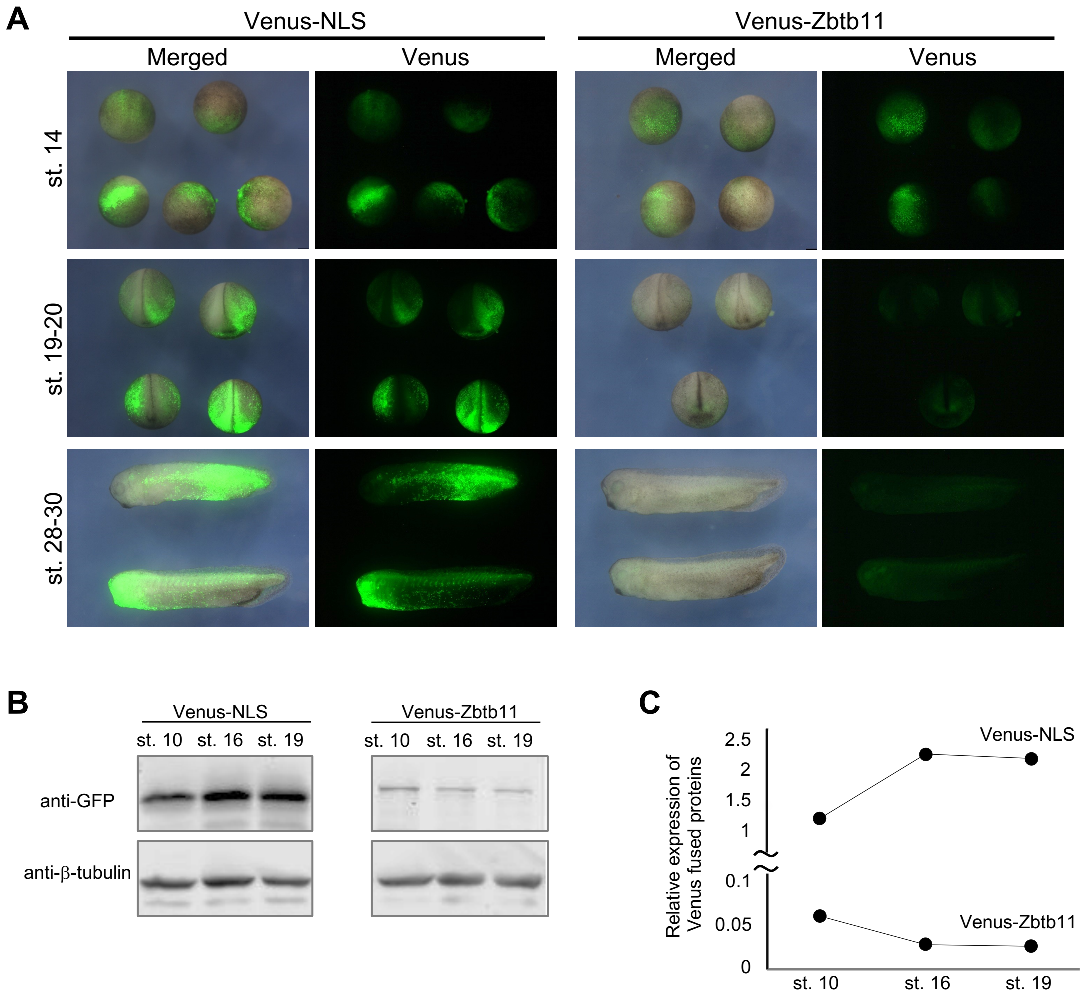

S4 Fig. Protein levels of Venus-Zbtb11 expressed in embryos.

(A) Fluorescence observation of Venus-NLS and Venus-Zbtb11. mRNA for Venus-NLS or Venus-Zbtb11 was injected into any one of the blastomeres at the 4-cell stage and fluorescence was observed at stages 14, 19–20, and 28–30. There are no differences in the fluorescence intensity between injected regions, indicating that the protein stability of Venus-Zbtb11 does not depend on tissue type. Merged, fluorescence images merged with bright-field images; Venus, fluorescence images. (B,C) Comparison of protein expression levels between Venus-NLS and Venus-Zbtb11. mRNA for Venus-NLS or Venus-Zbtb11 was injected into both blastomeres at the 2-cell stage. Lysates were prepared at stages 10, 16, and 19 and subjected to western blotting using anti-GFP and anti-β-tubulin antibodies. (B) Western blotting of Venus-NLS and Venus-Zbtb11. Upper panels, bands of Venus-NLS and Venus-Zbtb11; lower panels, bands of β-tubulin as a loading control. (C) Expression levels of Venus-NLS and Venus-Zbtb11. The relative expression level was obtained by dividing the band intensity of Venus-NLS or Venus-Zbtb11 by that of β-tubulin. Stages (st.) are indicated, respectively.
