# Supplementary material for: Zbtb11 interacts with Otx2 and patterns the anterior neuroectoderm in Xenopus
Source: PLoS One. 2024 Jul 31;19(7):e0293852. doi: 10.1371/journal.pone.0293852 (PMC11290676; doi:10.1371/journal.pone.0293852)
Supplement: S5 Fig — mRNA for zbtb11-MO target sequences fused with eGFP (Zbtb11-ATG-eGFP construct) was injected into two blastomeres at the 2-cell stage and then zbtb11-MO or control-MO was injected into all blastomeres at the 4-cell stage. Lysates were prepared from gastrula embryos and subjected to western blotting with anti-GFP and anti-β-tubulin antibodies. Translation of zbtb11-eGFP mRNA was blocked by zbtb11-MO injection compared to control-MO-injected or uninjected samples. Amounts of injected mRNA for Zbtb11-ATG-eGFP (pg/embryo), 500; injected MOs (pmol/embryo), 1. (PDF) [file pone.0293852.s005.pdf]

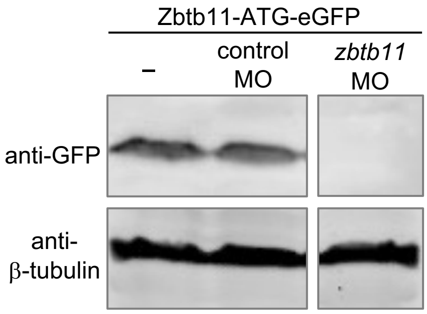

S5 Fig. Specificity of *zbtb11*-morpholino oligo (MO).

mRNA for *zbtb11*-MO target sequences fused with eGFP (Zbtb11-ATG-eGFP construct) was injected into two blastomeres at the 2-cell stage and then *zbtb11*-MO or control MO was injected into all blastomeres at the 4-cell stage. Lysates were prepared from gastrula embryos and subjected to western blotting with anti-GFP and anti-*β*-tubulin antibodies. Translation of *zbtb11*-eGFP mRNA was blocked by *zbtb11*-MO injection compared to control MO-injected or uninjected samples. Amounts of injected mRNA for Zbtb11-ATG-eGFP (pg/embryo), 500; injected MOs (pmol/embryo), 1.
