# Supplementary material for: Zbtb11 interacts with Otx2 and patterns the anterior neuroectoderm in Xenopus
Source: PLoS One. 2024 Jul 31;19(7):e0293852. doi: 10.1371/journal.pone.0293852 (PMC11290676; doi:10.1371/journal.pone.0293852)
Supplement: S6 Fig — Original images for (A) Fig 7A–7C, (B) Fig 8A and 8B, (C) Fig 9B, (D) S3 Fig, (E) S4B Fig and (F) S5 Fig are shown. See the corresponding figure legends for details. Boxes indicate the cropped images presented in the figures. M, protein size markers. Molecular masses (kDa) of the protein size markers are as indicated. (PDF) [file pone.0293852.s006.pdf]

**A**

# Fig 7A

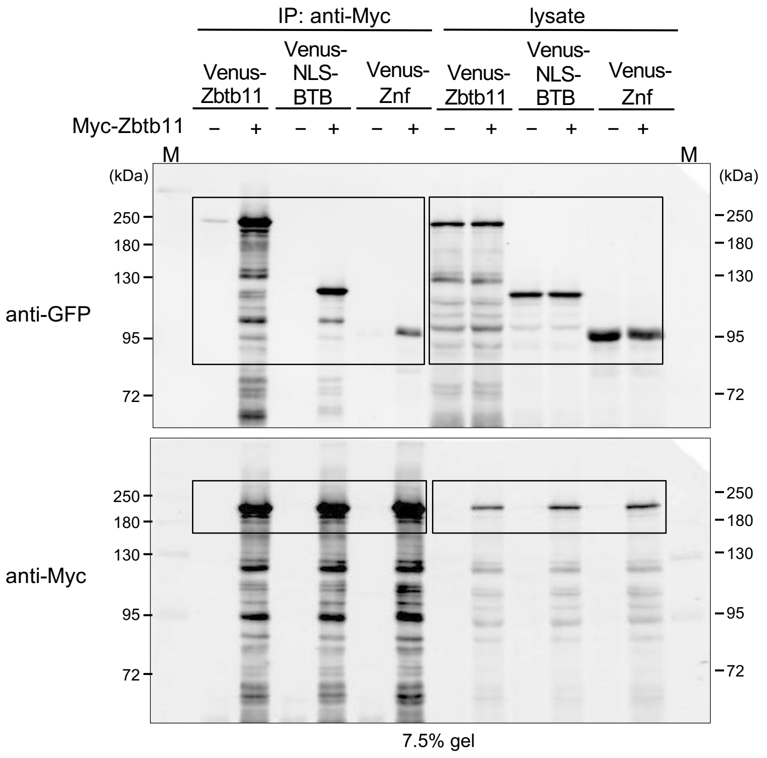


# Fig 7B

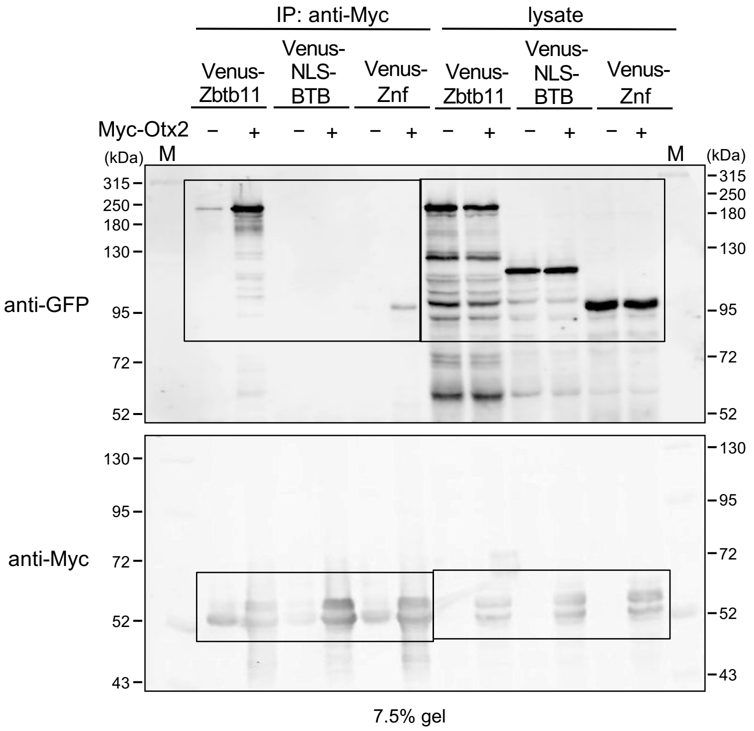

**A (continued)**

# Fig 7C

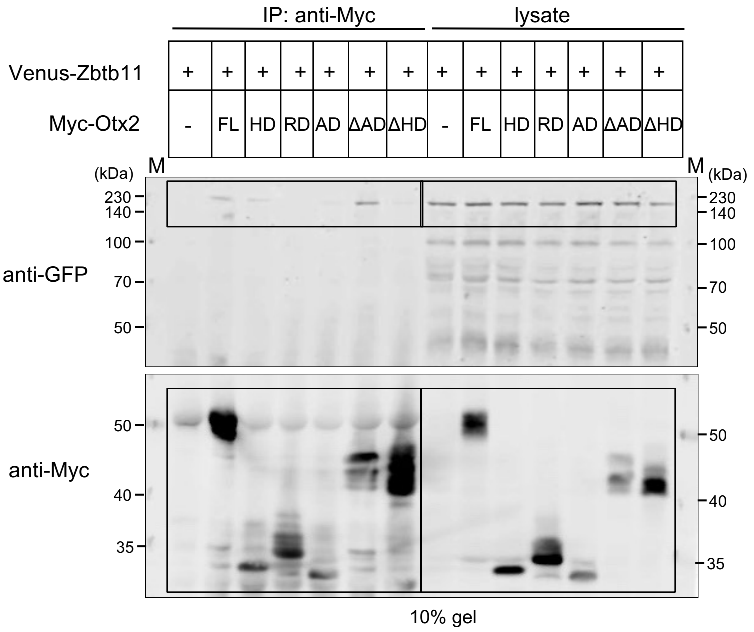

**B**

# Fig 8A

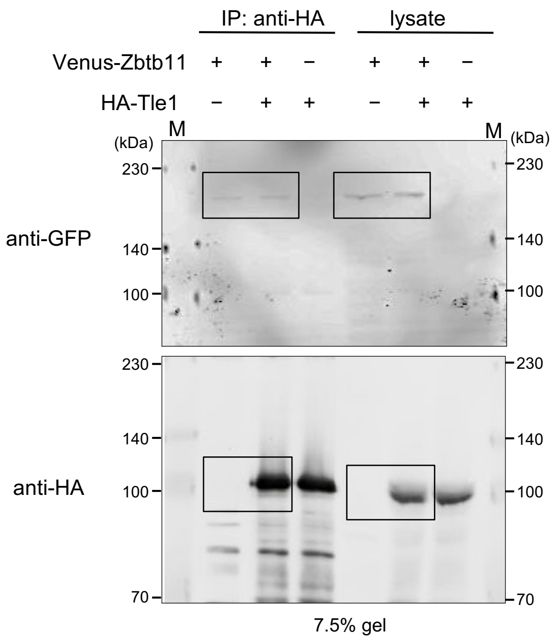


# Fig 8B

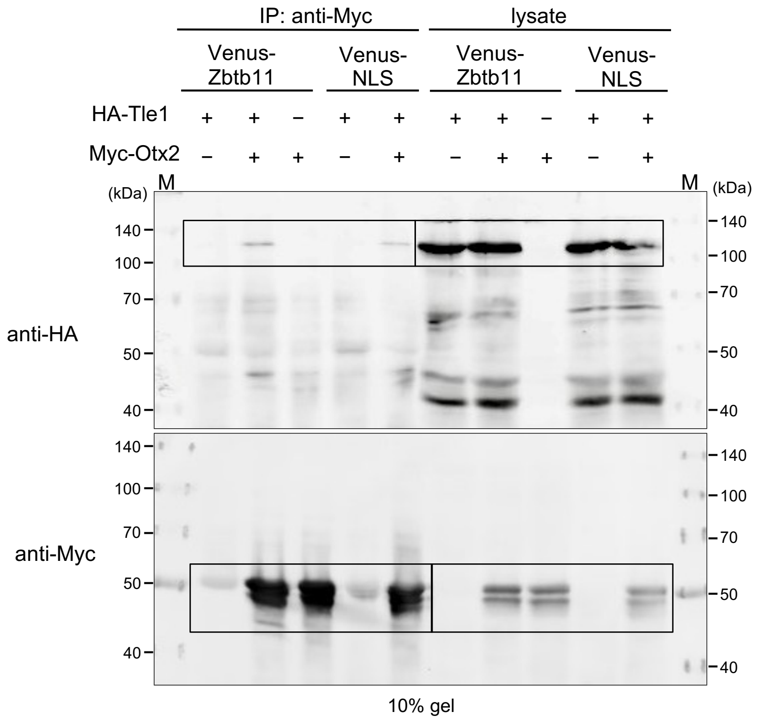

**C**

# Fig 9B

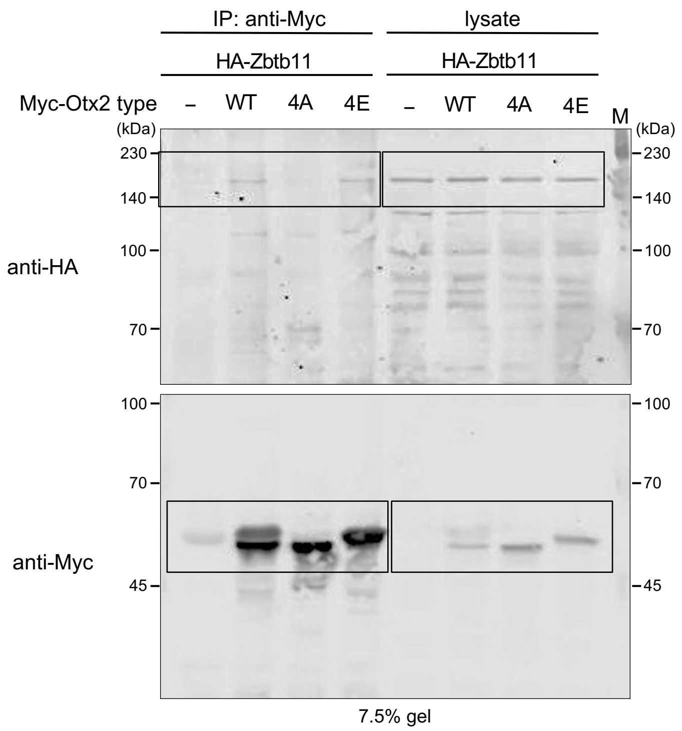
IP: anti-Myc
lysate
HA-Zbtb11
HA-Zbtb11
Myc-Otx2 type
–
WT
4A
4E
–
WT
4A
4E
M
(kDa)
(kDa)
230
140
100
70
anti-HA
100
70
45
anti-Myc
7.5% gel

**D**

## S3A Fig

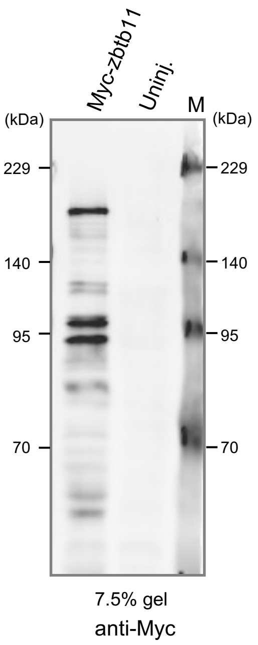


## S3B Fig

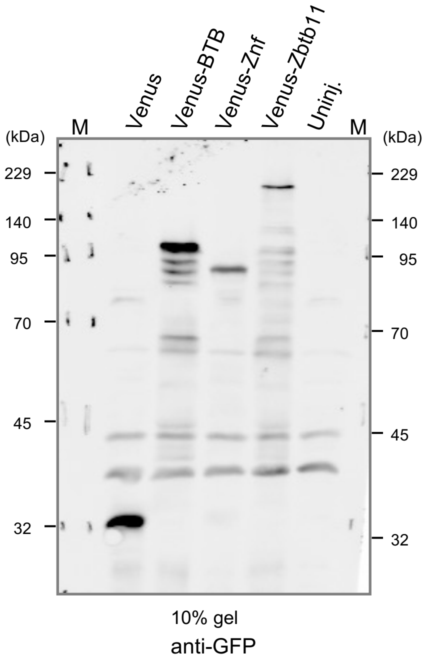

**E**

# S4B Fig

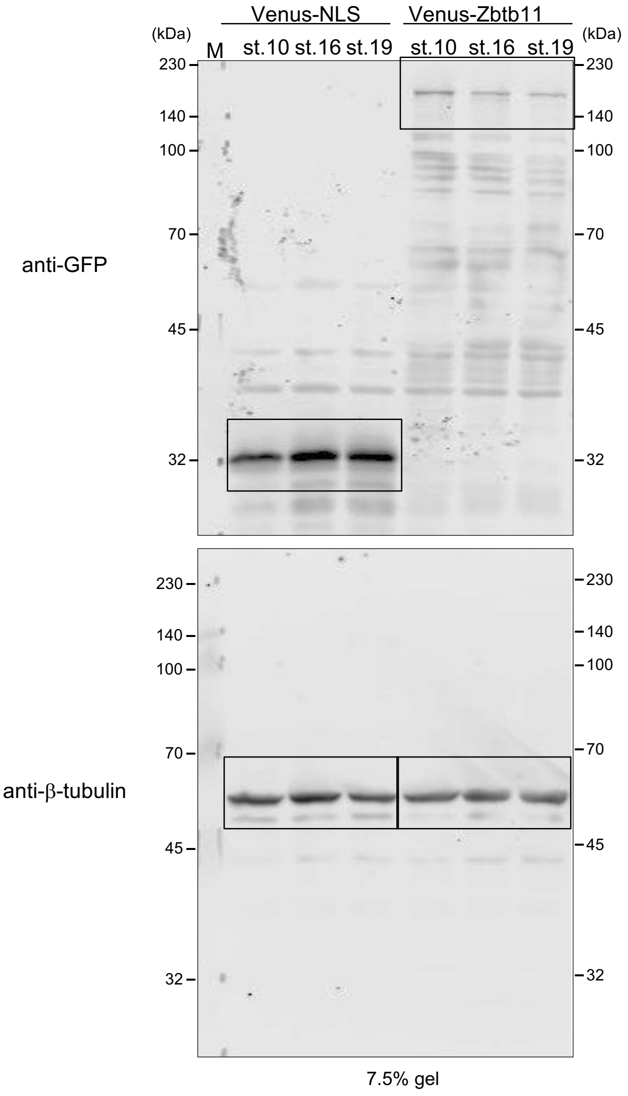

Venus-NLS
Venus-Zbtb11
(kDa)
M
st.10 st.16 st.19
st.10 st.16 st.19
(kDa)
230
140
100
70
45
32
anti-GFP
anti-β-tubulin
7.5% gel

**F**

S5 Fig

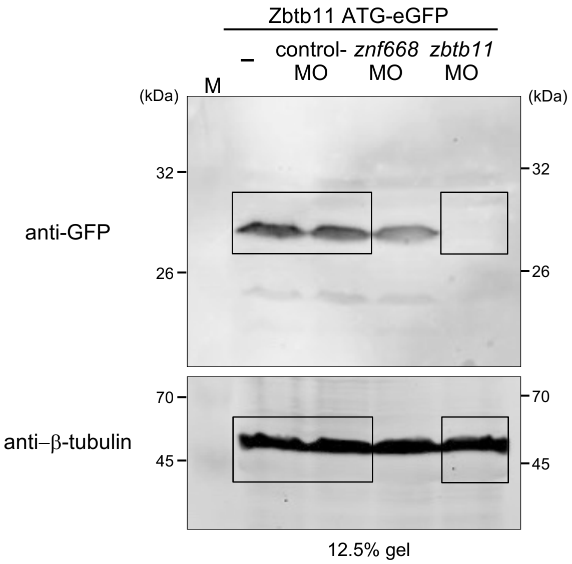


**S6 Fig. Original, uncropped and minimally adjusted images of western blots.**

Original images for (A) Fig 7A-C, (B) Fig 8A and 8B, (C) Fig 9B, (D) S3 Fig, (E) S4B Fig, and (F) S5 Fig are shown. See the corresponding figure legends for details. Boxes indicate the cropped images presented in the figures. M, protein size markers. Molecular masses (kDa) of the protein size markers are as indicated.
