# Supplementary material for: Zbtb11 interacts with Otx2 and patterns the anterior neuroectoderm in Xenopus
Source: PLoS One. 2024 Jul 31;19(7):e0293852. doi: 10.1371/journal.pone.0293852 (PMC11290676; doi:10.1371/journal.pone.0293852)
Supplement: S1 Table — (PDF) [file pone.0293852.s007.pdf]

S1 Table. The list of plasmid constructs.

Plasmid names	Cloning sites	Vectors	Comments
pCSf107_Zbtb11_T	*Bam*HI, *Xba*I	pCSf107_mT	aa 1-1118 (full length); Zbtb11.S (*X. laevis*), XM_018249724
pCSf107_Venus-Zbtb11_T	*Bam*HI, *Xba*I	pCSf107_Venus_mT	Replaced pCSf107-Zbtb11_T
pCSf107_Venus-BTB_T	*Bam*HI, *Xba*I	pCSf107_Venus_mT	aa 1-580
pCSf107_Venus-ZF_T	*Bam*HI, *Xho*I	pCSf107_Venus_mT	aa 622-1118
pCSf107_Venus-NLS-BTB_T	*Bam*HI, *Sac*II	pCSf107_Venus-NLS_mT	Replaced pCSf107_Venus-BTB_T
pCSf107_Zbtb11-ATG-eGFP_T	*Bam*HI, *Xba*I	pCSf107_mcs2-eGFP-C2mT	*zbtb11*-MO target sequences (*X. tropicalis*)
pCSf107_pax6_T	*Sfu*I, *Not*I	pCSf107_mT	Replaced pCS105-pax6
pCSf107_Myc-Zbtb11_T	*Bam*HI, *Asc*I	pCSf107_MycmT	Replaced pCSf107-Zbtb11_T
pCSf107_HA-Zbtb11_T	*Bam*HI, *Asc*I	pCSf107_HAmT	Replaced pCSf107-Zbtb11_T
pCSf107_MycOtx2FL_T	*Bam*HI, *Xba*I	pCSf107_MTmT	aa 1-288 (full length) Otx2.S (*X. laevis*), NP_001084160
pCSf107_MycOtx2HD_T	*Bam*HI, *Xba*I	pCSf107_MTmT	aa 1-96
pCSf107_MycOtx2RD_T	*Bam*HI, *Xba*I	pCSf107_MTmT	aa 97-184
pCSf107_MycOtx2AD_T	*Bam*HI, *Xba*I	pCSf107_MTmT	aa 185-288
pCSf107_MycOtx2ΔAD_T	*Bam*HI, *Xba*I	pCSf107_MTmT	aa 1-184
pCSf107_MycOtx2ΔHD_T	*Bam*HI, *Xba*I	pCSf107_MTmT	aa 97-288
pCSf107_HATle1_T	*Age*I, *Xba*I	pCSf107_4HAmT	Replaced pCSf107-Tle1
pCSf107_Otx2_T	*Bam*HI, *Xba*I	pCSf107_mT	Untagged Otx2
pCSf107_MycOtx2-4A_T	*Bam*HI, *Xba*I	pCSf107_MTmT	T115A, S116A, S132A, S158A
pCSf107_MycOtx2-4E_T	*Bam*HI, *Xba*I	pCSf107_MTmT	T115D, S116E, S132E, S158E
pCSf107_Otx2-4A_T	*Bam*HI, *Xba*I	pCSf107_mT	T115A, S116A, S132A, S158A
pCSf107_Otx2-4E_T	*Bam*HI, *Xba*I	pCSf107_mT	T115D, S116E, S132E, S158E

Note: the postfix “_T” indicates the presence of SP6/T7 terminators at the end of the transcribed region.
