# Supplementary material for: Zbtb11 interacts with Otx2 and patterns the anterior neuroectoderm in Xenopus
Source: PLoS One. 2024 Jul 31;19(7):e0293852. doi: 10.1371/journal.pone.0293852 (PMC11290676; doi:10.1371/journal.pone.0293852)
Supplement: S2 Table — (PDF) [file pone.0293852.s008.pdf]

S2 Table. The list of cutting sites and RNA polymerases for the in vitro transcription of anti-sense RNA probe.

Gene or plasmid names	Cutting sites	RNA polymerase
En-2	*Bam*HI	T3
pCSf107_Otx2-WT_T	*Bam*HI	T7
pCS107BSX_rax	*Xho*I	SP6
pCSf107_pax6_T	*Sal*I	T7
pBS SK(-)gbx2	*EcoR*I	T3
BS4A 3(X pax2-2a)	*EcoR*I	T3
pBSK_xcg1	*Not*I	T3
